# Supplementary material for: A bibliometric analysis of geographic disparities in the authorship of leading medical journals
Source: Commun Med (Lond). 2023 Dec 11;3:178. doi: 10.1038/s43856-023-00418-2 (PMC10713520; doi:10.1038/s43856-023-00418-2)
Supplement: Supplementary file 1 — Supplementary Information [file 43856_2023_418_MOESM1_ESM.pdf]

A Bibliometric Analysis of Geographic Disparities in the Authorship of Leading Medical Journals

Supplementary Information

Oscar Brück[1]

1 *Hematoscope Lab, Comprehensive Cancer Center & Center of Diagnostics, Helsinki University Hospital, Helsinki, Finland & Department of Oncology, University of Helsinki, Helsinki, Finland*

Supplementary Table 1. Domestic Self-Citation Index Calculation by Country.

Country	#Domestic citations of one country	#All citations of one country	%Domestic citations	#Citations of other countries	#Citations from other countries	#Citations of other countries / #Citations from other countries	Domestic Self-Citation Index
China	3870	84535	4.6	80665	12090	15	0.31
USA	82159	219717	37.4	137558	226603	164.7	0.23
Japan	4363	32204	13.5	27841	20258	72.8	0.19
Bangladesh	372	2136	17.4	1764	1811	102.7	0.17
Brazil	4196	19136	21.9	14940	19968	133.7	0.16
India	2044	14941	13.7	12897	12773	99	0.14
Germany	1938	50300	3.9	48362	15690	32.4	0.12
Australia	2400	40200	6	37800	21661	57.3	0.10
South Korea	1326	16809	7.9	15483	12221	78.9	0.10
Italy	2056	42986	4.8	40930	19765	48.3	0.10
Saudi Arabia	507	4726	10.7	4219	4654	110.3	0.10
UK	23539	86864	27.1	63325	183036	289	0.09
France	1513	34587	4.4	33074	15900	48.1	0.09
South Africa	2189	9507	23	7318	20126	275	0.08
Canada	1700	42208	4	40508	19687	48.6	0.08
Sweden	1062	17647	6	16585	12698	76.6	0.08
Denmark	810	13798	5.9	12988	10110	77.8	0.08
Spain	2792	28548	9.8	25756	33401	129.7	0.08
Netherlands	2513	31078	8.1	28565	34781	121.8	0.07
Singapore	837	6951	12	6114	11315	185.1	0.07
Pakistan	413	3538	11.7	3125	6299	201.6	0.06
Israel	661	6377	10.4	5716	10390	181.8	0.06
Norway	915	8782	10.4	7867	14737	187.3	0.06
Thailand	682	4475	15.2	3793	10703	282.2	0.05
Ireland	335	6289	5.3	5954	6014	101	0.05
Switzerland	1291	21908	5.9	20617	23425	113.6	0.05
Finland	713	6823	10.4	6110	13211	216.2	0.05
Belgium	1355	15135	9	13780	25801	187.2	0.05
Austria	728	9236	7.9	8508	15439	181.5	0.04
New Zealand	898	5760	15.6	4862	17484	359.6	0.04
Kenya	383	2817	13.6	2434	7869	323.3	0.04
Greece	252	7806	3.2	7554	6031	79.8	0.04

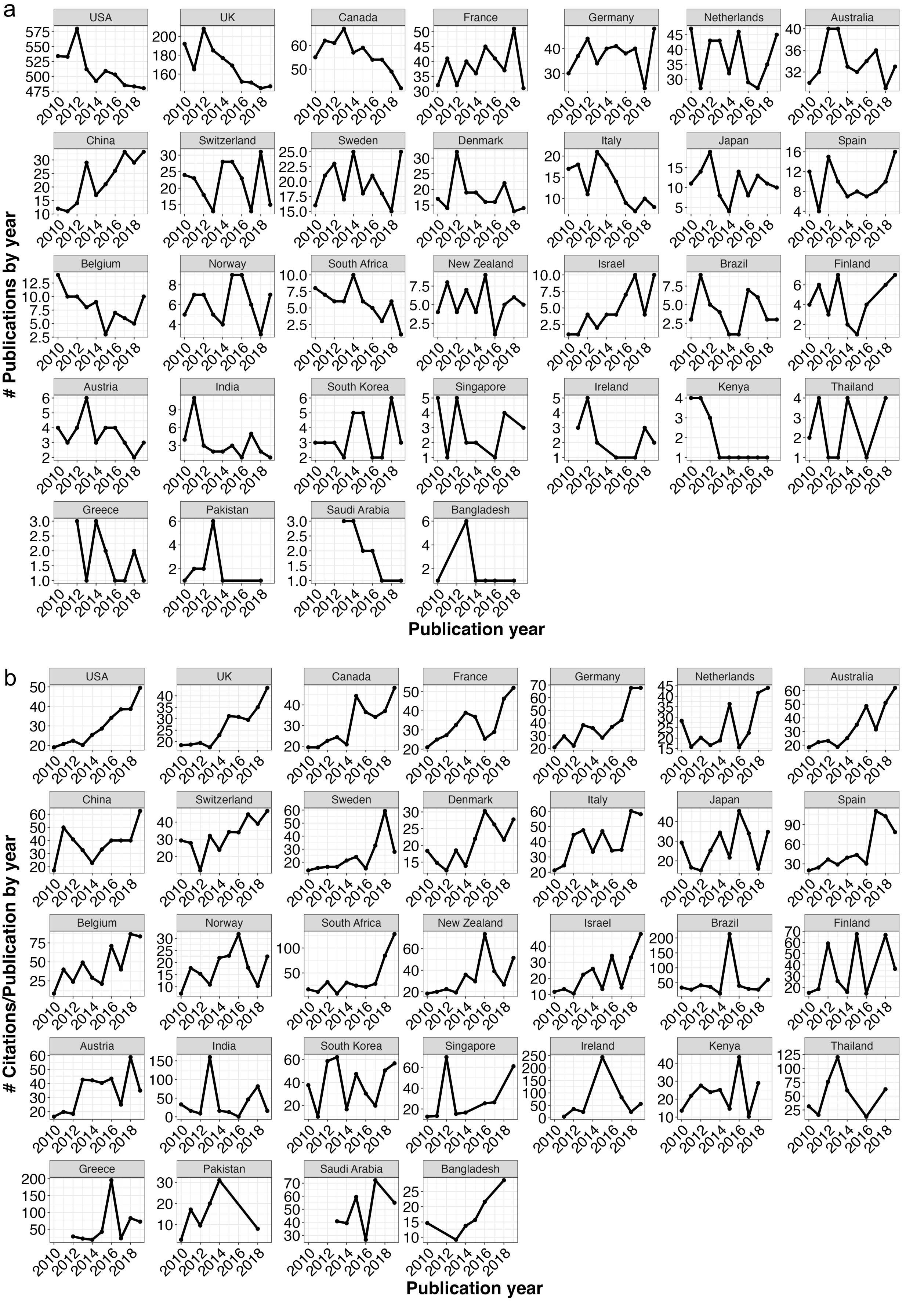

Supplementary Figure 1. a. Line plot illustrating the number of publications and b. citations per publication by their publishing year for the top 32 most productive countries. Countries are arranged by their total publication count between 2010-2019 in descending order.

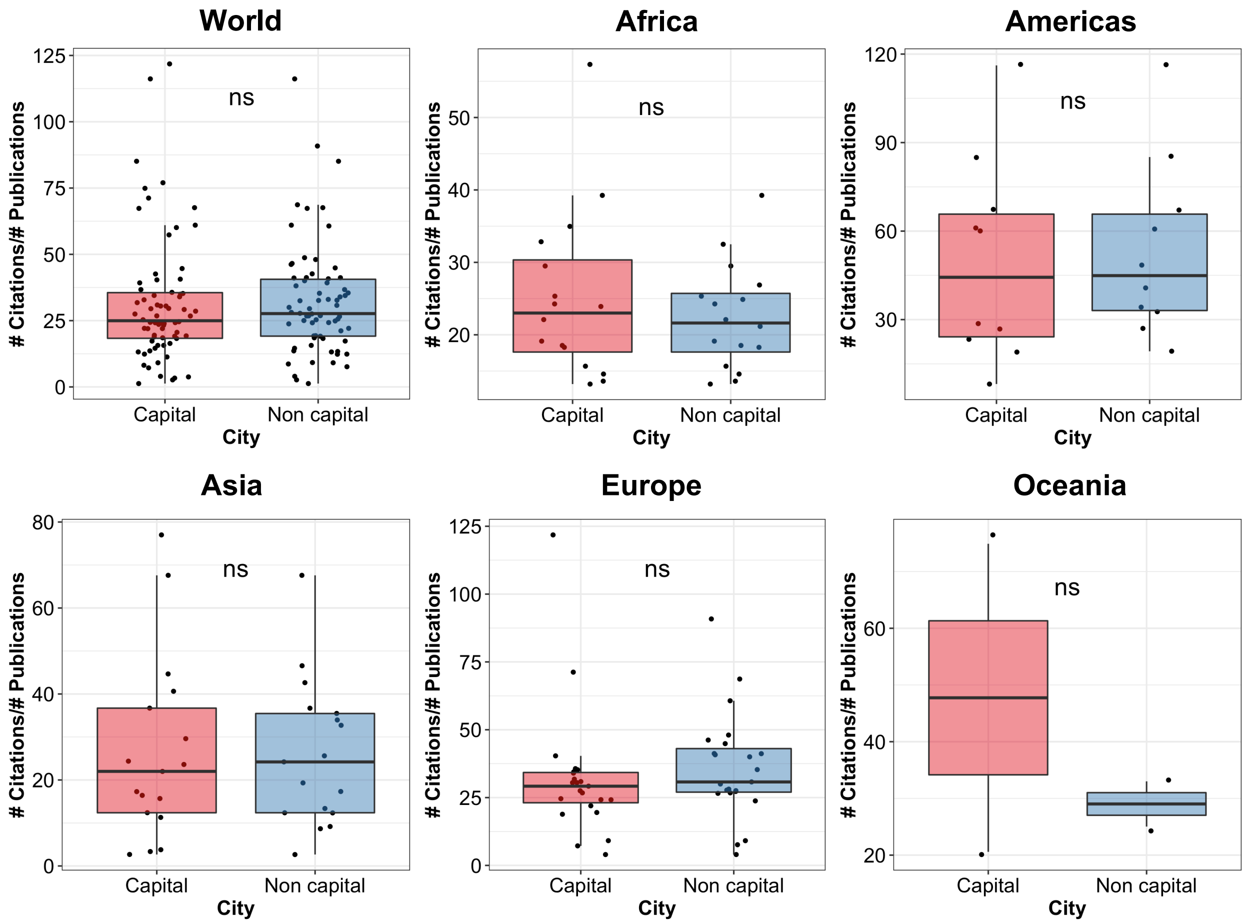

Supplementary Figure 2. Box plots illustrating the number of citations per publication in capital vs. non-capital cities in the every continent (World) and by distinct continents (Paired Wilcoxon test). The box covers the interquartile interval, and the line that split the box in two is the median. The lower and upper sides of the box are the lower and upper quartiles.

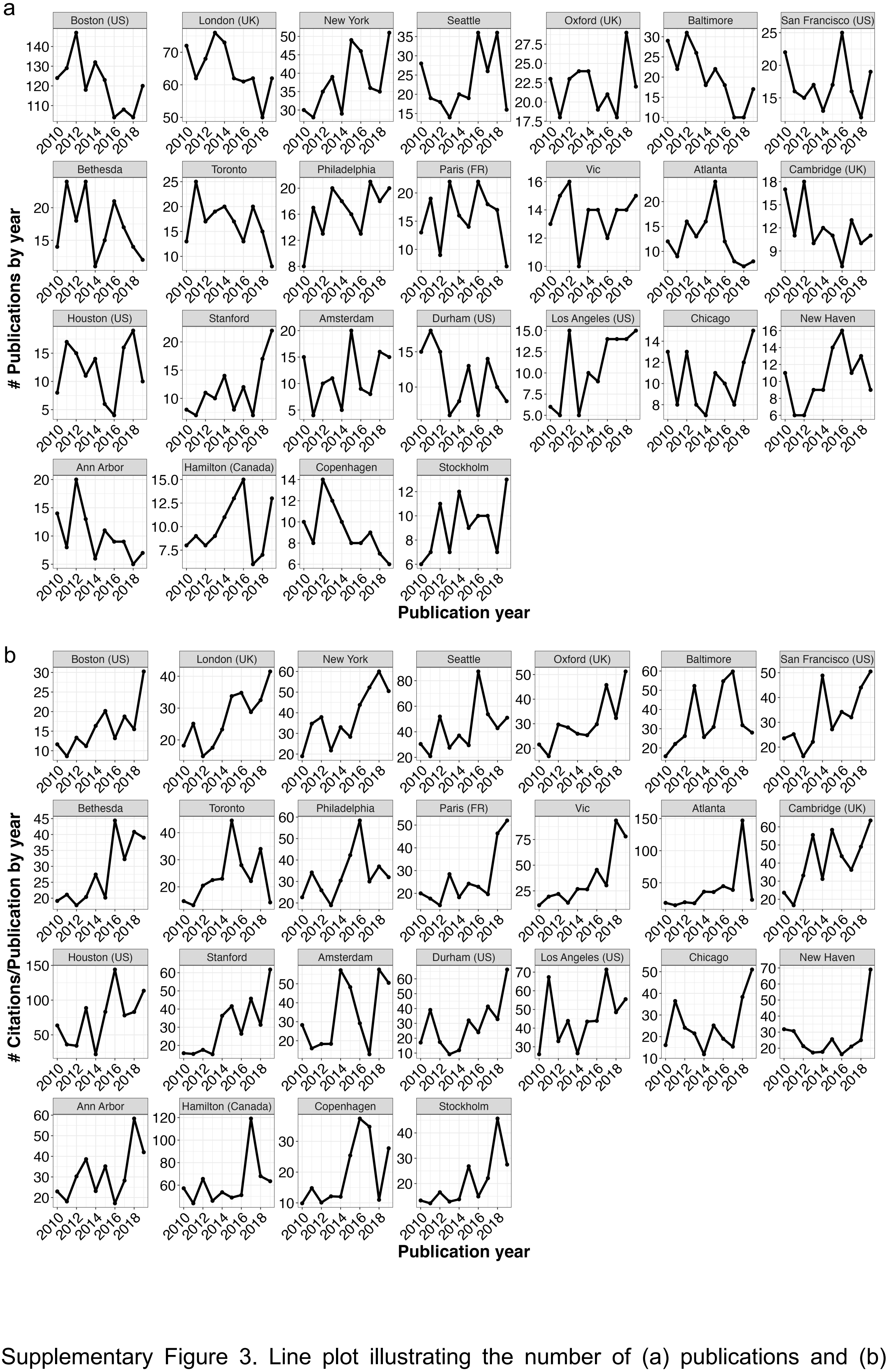

Supplementary Figure 3. Line plot illustrating the number of (a) publications and (b) yearly-averaged citations per publication by their publishing year for the top 25 most productive cities. Cities are arranged by their total publication count between 2010-2019 in descending order.

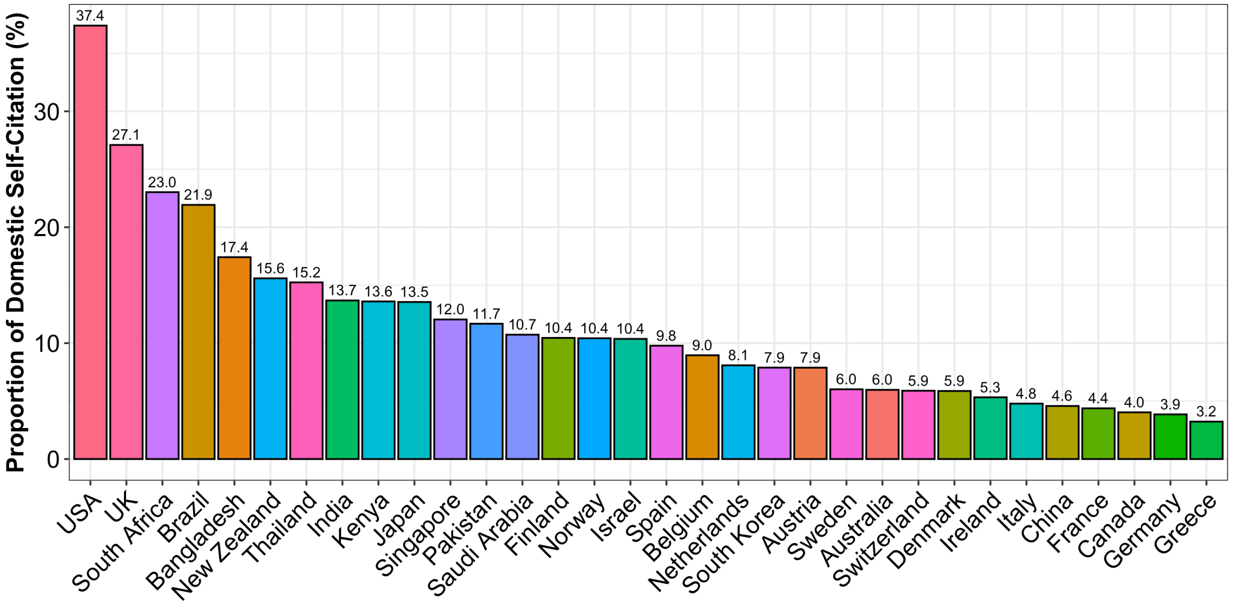

Supplementary Figure 4. Bar plot illustrating the proportion of domestic citations of all (domestic + international) citations by citing country.

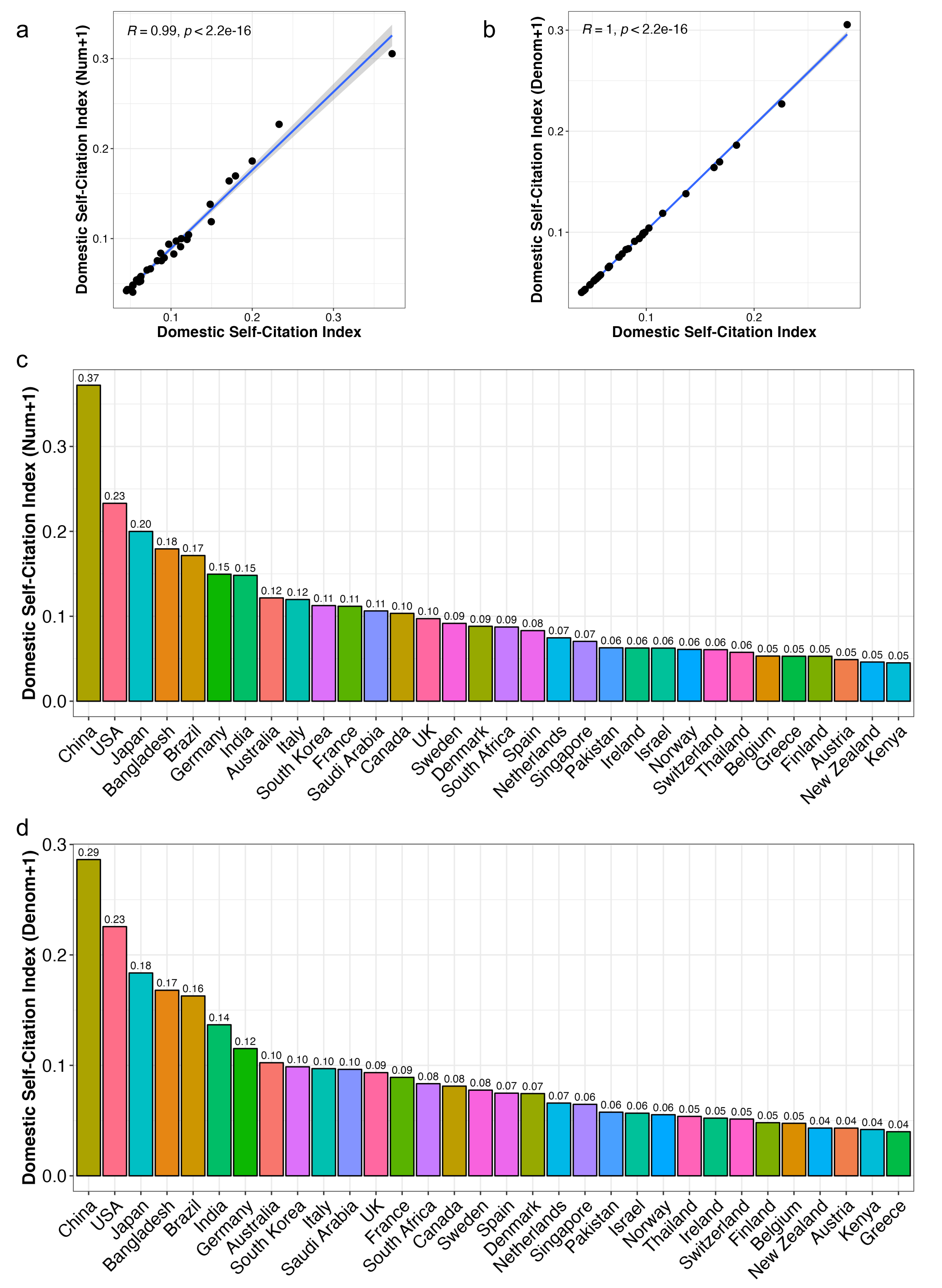

Supplementary Figure 5. Tests to confirm the robustness of the Domestic Self-Citation Index. (a) Correlation plots between the original Domestic Self-Citation Index and itself after adding 1 to its numerator or (b) to its denominator. The shaded area represents the 95% confidence interval of the regression model. (c) Bar plot illustrating the proportion of how much other countries have cited any given country in relation to how much they have cited all other countries, when adding 1 to the numerator or (d) to the denominator of the Domestic Self-Citation Index.
